# Association between Skin Carotenoid Score Measured with Veggie Meter^®^ and Adherence to the Mediterranean Diet among Adolescents from Southern Italy

**DOI:** 10.3390/nu15234920

**Published:** 2023-11-25

**Authors:** Giovanna Caparello, Giovanni Dongionny Groccia, Fabrizio Ceraudo, Mirko Cesario, Renzo Bonofiglio, Giuseppina Augimeri, Daniela Bonofiglio

**Affiliations:** 1 Department of Pharmacy, Health and Nutritional Sciences, University of Calabria, Arcavacata di Rende, 87036 Cosenza, Italy; caparello.giovanna@gmail.com (G.C.); giovannigroccia20798@gmail.com (G.D.G.); fabrizio.cer96@gmail.com (F.C.); mirkocesario@gmail.com (M.C.); giuseppina.augimeri@unical.it (G.A.); 2 Department of Nephrology, Dialysis and Transplantation, “Kidney and Transplantation” Research Centre, Annunziata Hospital, 87100 Cosenza, Italy; rbonofi@gmail.com; 3 Centro Sanitario, University of Calabria, Arcavacata di Rende, 87036 Cosenza, Italy

**Keywords:** carotenoids, fruit intake, vegetable intake, Veggie Meter^®^, Mediterranean diet, adolescents

## Abstract

The Veggie Meter^®^ (Longevity Link Corporation, Salt Lake City, UT, USA), is a new portable device for the non-invasive and rapid detection of skin carotenoid content, which represents an acceptable biomarker for the evaluation of fruit and vegetable (FV) intake. FVs are important components of a healthy diet, including the Mediterranean Diet (MD), which is a plant-based dietary pattern. Here, we evaluated the adherence to the MD via the administration of two online food questionnaires, and we measured the skin carotenoid content using the Veggie Meter^®^ in a cohort of 498 healthy adolescents (233 males and 265 females) from Southern Italy. Using KIDMED and the MD Pyramid tests to assess the adherence to the MD, we found an average adherence (5.43 ± 2.57 and 7.20 ± 1.93, respectively) to the MD in our sample population. Moreover, we observed that the skin carotenoid score was 364.75 ± 98.29, which was within the normal range and inversely related to the BMI (r = −0.1461, *p* = 0.0011). Similar results were observed when the population was categorized by sex. Interestingly, we demonstrated, for the first time, a positive correlation between the carotenoid score and the adherence to the MD assessed using both the KIDMED and MD Pyramid tests in the total population (r = −0.2926, *p* < 0.0001 and r = −0.1882, *p* < 0.0001, respectively). The same direct correlation was found in adolescents according to their sex. Our findings highlight the potential of the Veggie Meter^®^ as a feasible and promising tool for evaluating adherence to the MD and, ultimately, to promote healthy eating habits among adolescents.

## 1. Introduction

Fruits and vegetables (FVs) are important components of a healthy diet. Based on the data reported by the World Health Organization (WHO), around 3.9 million deaths were associated with inadequate FV intake in 2017 worldwide [1]. In addition, a low intake of FVs has been linked to the development of chronic non-communicable diseases [1,2,3,4,5]. Indeed, FV is characterized by a low content of saturated fats, adequate fiber content, and a high amount of vitamins along with a broad spectrum of non-nutrient molecules such as plant sterols and flavonoids, which exert important health benefits [6,7,8,9,10]. Carotenoids, a group of natural fat-soluble pigments of the polyene type produced by plants and algae, are one of the most representative classes of active compounds found in FV [11,12,13], influencing the color of FV as well as their beneficial actions [14,15,16]. Although more than 700 carotenoids occur in nature, only 6, including β-carotene, β-cryptoxanthin, α-carotene, lycopene, lutein, and zeaxanthin, have been found in the diet and represent more than 95% of total blood carotenoids [17]. Since humans are not able to endogenously produce carotenoids [18], they primarily obtain these biomolecules via dietary sources, with FV serving as a primary contributor to their intake [19]. Thus, the total serum carotenoid levels represent the best biomarker of FV intake from the diet [20,21]. The evaluation of carotenoids in the body is based on the collection of plasma or serum samples and subsequent analysis via high-performance liquid chromatography (HPLC) with mass spectrometry (MS) [22,23]. However, this serum evaluation of carotenoid levels is an invasive and expensive method. In addition, the short half-life of carotenoids reduces the accuracy of the measurements. Therefore, more suitable methods for the evaluation of carotenoids are developing.

In this scenario, the assessment of skin carotenoids using the Veggie Meter^®^ is becoming an interesting accurate tool to evaluate the carotenoid content in the human body. In fact, the Veggie Meter^®^ is an innovative portable device that uses reflection spectroscopy and pressure-mediated reflection to determine the content of skin carotenoids in the fingertip in a single reading of 15 s. The Veggie Meter^®^ is connected to a computer, which shows the skin carotenoid score from 0 to 800 [24], allowing evaluation in a less expensive and complex procedure compared to other traditional analytic methods used for the skin carotenoid measurement.

Several studies have reported that the number of carotenoids in the skin is highly correlated with the serum carotenoid concentration and reflects the FV dietary intake in the previous 2–4 weeks [25]. Thus, Veggie Meter^®^ represents a feasible, reliable, and potentially valid measure of FV consumption [26,27,28,29]. Although high FV consumption is recommended for healthy benefits, a reduced intake of FV has been observed in the population, including adolescents [30,31,32]. This is alarming, as dietary patterns established during childhood and adolescence are likely to continue into adulthood [33]. In this context, different initiatives have been taken to promote and evaluate FV consumption among children and adolescents in schools, including developing nutritional programs based on dietary patterns that suggest a high FV intake. A recent pilot study has been carried out in a school children population to assess the intake of FV via Veggie Meter^®^ measurement, which resulted in an objective evaluation of FV consumption with respect to their self-reported intake [34]. Regarding the promotion of FV consumption, the Mediterranean diet (MD) is considered one of the healthiest dietary patterns based on the consumption of plant-origin foods [35]. The term MD describes the eating habits and lifestyle of countries from the Mediterranean Basin, mostly Greece, Cyprus, and Southern Italy, where the food cultures of ancient civilizations developed. MD model is based on the predominant consumption of plant foods such as cereals, fruits, vegetables, legumes, nuts, and seeds; a regular intake of olive oil, as the main source of fat; a moderate consumption of white meat, fish, seafood, low-fat dairy, and red wine; and a low intake of red meat, processed meat products, and sweets [36]. Thus, according to the MD recommendation, every meal should include two or more servings of vegetables and three or more servings of fruits. Several food frequency questionnaires are currently available to assess adherence to the MD, although the data do not always have acceptable validity and good reliability.

The Mediterranean Diet Quality Index (KIDMED) is one of the most frequently used scores to assess adherence to the MD pattern among children and adolescents in different countries from the Mediterranean and non-Mediterranean areas [37,38,39,40,41]. In our recent investigation, we used the KIDMED test to evaluate adherence to the MD in a cohort of adolescents living in a Southern Italy region [42]. According to other studies carried out in the Mediterranean countries [43,44], we found, in our population sample, a medium adherence to the MD, and, specifically, the compliance rates for the intake of more vegetables a day and a second fruit each day were definitively outside the recommendations [42], suggesting the need to promote the beneficial effects of FV intake.

Another tool used to investigate the food frequency consumption among the young population is represented by the National Youth Physical Activity and Nutrition Study (NYPANS) questionnaire, which measured the prevalence of determinants related to physical activity and nutrition among high school students [45].

Here, we evaluated the adherence to the MD via the administration of two online food questionnaires, and we measured the content of skin carotenoids using the Veggie Meter^®^ in a population of Calabrian healthy school adolescents enrolled in the context of the Pre.Di.Re study.

## 2. Materials and Methods

### 2.1. Study Design

As part of the scientific project “Pre.Di.Re” (Prevenzione delle malattie renali) of the ASIT (Associazione Sud Italia Trapiantati), a total of 498 subjects (233 males and 265 females) aged between 13 and 19 years were recruited among the students from the Scientific High School “G. B. Scorza” of Cosenza in Southern Italy. The data were collected from January 2023 to April 2023. A comprehensive explanation of this study’s objectives was presented to all participants, and written informed consent was obtained from both the participants and their parents as a prerequisite before their enrollment in this Pre.Di.Re study. Participants were asked to complete the questionnaires to assess the MD adherence and were subjected to study visits and measurements of skin carotenoids. The rationale of the research project and the adequacy of the protocol, according to the guidelines laid down in the Declaration of Helsinki, were approved by the Ethics Committee of the University of Calabria, Italy (UCALPRG Prot. n. 0075402 17 October 2022).

### 2.2. Anthropometric Parameters

Trained personnel gathered anthropometric data using established and standardized procedures [42]. Participants’ weights were determined using the KERN MPC250K100 M scale, with a load capacity of 250 kg and an accuracy of 100 g. Height was determined using a Seca stadiometer, with a maximum capacity of 220 cm and an accuracy of 1 mm. Body mass index (BMI) and BMI Z-score were calculated as previously reported [46].

### 2.3. Adherence to the Mediterranean Diet in KIDMED and Pyramid Questionnaires

Adherence to the MD was assessed when administering the validated Mediterranean Diet Quality Index (KIDMED) test and the MD Pyramid questionnaire to all participants, which was used for the first time in this study.

The KIDMED comprises 16 items, with 4 questions reflecting unfavorable dietary habits (consumption of fast food, baked goods, sweets, and skipping breakfast), scored with a value of −1 each, and 12 questions denoting a positive connotation (consumption of oil, fish, fruits, vegetables, cereals, nuts, pasta or rice, dairy products, and yogurt), scored with a value of +1 each. The resulting KIDMED score ranges from <0 to ≤12, with higher scores representing a higher adherence to the MD. Based on the degree of the KIDMED score, we classified our population sample into high (≥8 points), medium (4 to 7 points), and low (≤3 points) MD adherence [37].

The MD Pyramid questionnaire was designed adapting the NYPANS questionnaire (http://www.cdc.gov/healthyYouth/yrbs/pdf/nypans/2010nypans_questionnaire.pdf, accessed on 23 May 2022) and was made up of 18 multiple choice questions regarding frequency of food consumption, with only one correct answer per question, as reported in Table A1 of Appendix A.

Wrong questions do not give points. The resulting MD Pyramid score ranged from 0 to 18, with higher scores representing a higher adherence to the MD. Based on the degree of the MD Pyramid score, we classified our population sample into high (>12 points), medium (7 to 12 points), and low (<7 points) MD adherence (Table A1 in Appendix A).

### 2.4. Measurement of Skin Carotenoid Content

Skin carotenoid levels were measured using the Veggie Meter^®^ (Longevity Link Corporation, Salt Lake City, UT, USA), a spectroscopy-based device (http://www.longevitylinkcorporation.com/products.html, accessed on 18 September 2023), according to the manufacturer’s instructions. Briefly, the device’s calibration was performed with the provided dark and white reference materials prior to the data collection. Then, the skin carotenoid measurement was executed on the index finger of the non-dominant hand of each adolescent after hand washing in a single measurement mode. The subjects inserted their finger into the device applying a modest pressure in order to decrease the blood perfusion of the measured tissue volume, which might interfere with the measurement of skin carotenoid content. Moreover, the Veggie Meter^®^, using an algorithmic deconvolution adjustment, identified and corrected for individual concentrations of melanin. A computer analyzed the light that was reflected from the finger and provided a score on a spectral range from 0 to 800, with a higher score indicating higher skin carotenoid stores. Information relating to the subject such as age, sex, weight, and height was entered into the appropriate software.

### 2.5. Statistical Analysis

Data were reported as the mean and standard deviation (SD). Statistical differences were evaluated using parametric tests (Student’s *t*-test, One-way ANOVA with Bonferroni or Dunnett’s multiple comparison test, and Chi-square test) in GraphPad-Prism 7.00 software program. One-way ANOVA with Bonferroni was used in Table 1; Multivariate linear regression model was analyzed using STATA 17.0 in Table 2; Chi-square test was used in Table 3 and Table 4; One-way ANOVA with Bonferroni was used in Table 5 and Figure 2; Chi-square test was used in Supplementary Tables S1 and S2. One-way ANOVA with Dunnett’s multiple comparison test was used in Supplementary Tables S5 and S6 One-way ANOVA with Dunnett’s multiple comparison test was used in Supplementary Tables S5 and S6, and Chi-square test was used in Table 3 and Table 4 and Supplementary Tables S1 and S2. Association between variables was evaluated using Pearson’s linear correlation index in GraphPad-Prism 4 software program. Statistical significance was set at *p* < 0.05. 

## 3. Results

### 3.1. General Characteristic and Carotenoid Score of the Study Population

The general characteristics of the total study population and categorized by sex are shown in Table 1. The mean age of the total population (498 subjects), composed of 233 males and 265 females, was 16.66 ± 1.53 years. Normal weight (62.87 ± 12.51), height (168.32 ± 8.78), and BMI (22.10 ± 3.44) values were found in our population sample. As expected, significant changes were found in weight (68.91 ± 13.05 vs. 57.57 ± 9, *p* < 0.0001), height (174.58 ± 6.70 vs. 162.81 ± 6.36, *p* < 0.0001), BMI (22.55 ± 3.67 vs. 21.71 ± 3.18, *p* = 0.0001), and BMI Z-score (0.31 ± 0.99 vs. 0.31 ± 0.99, *p* = 0.03) in the adolescents categorized by sex. In addition, the carotenoid content evaluated using the Veggie Meter^®^ was within the normal range (364.75 ± 98.29) in the total population. Notably, males exhibited a significantly higher score compared to females (384.52 ± 94.17 vs. 347.37 ± 98.72, *p* < 0.0001).

**Figure 1:**
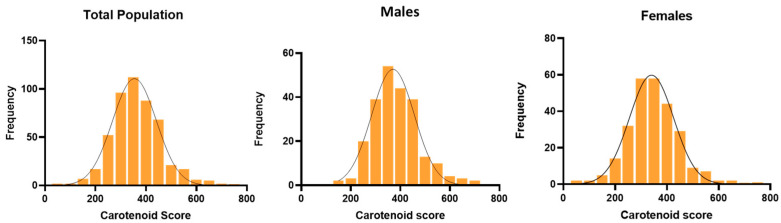
Distribution of the carotenoid scores with Veggie Meter^®^ in the total population and categorized by sex.

The distribution of carotenoid scores, ranging from 60 to 730, was reported in all students, males, and females (Figure 1).

### 3.2. Correlation between BMI and Carotenoid Score

**Figure 2:**
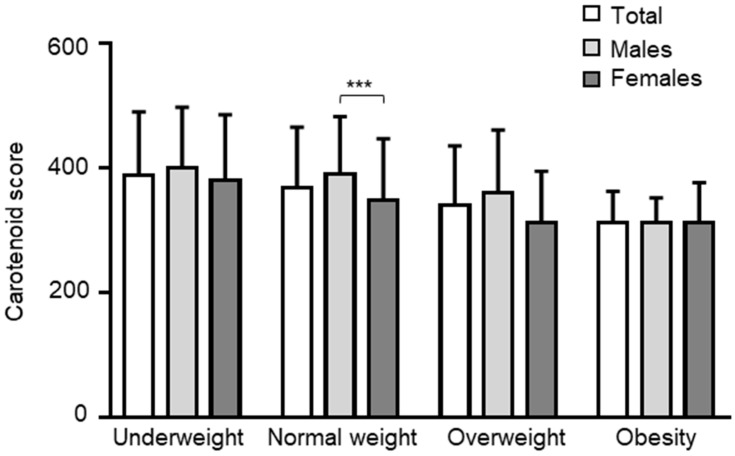
Carotenoid score in the total population and stratified by sex. *** *p* < 0.0001.

It has been demonstrated that deficiency of serum carotenoids represents a risk factor for overweight and obesity and that carotenoid supplementation in the diet is significantly correlated with reduced BMI in overweight or obese subjects [47]. In order to evaluate the correlation between BMI and the skin carotenoid content, we first divided the population into quartiles based on the BMI values and assessed the carotenoid content measured using the Veggie Meter^®^ in the population. Interestingly, by categorizing the population by sex, a higher carotenoid score was found in males than females within the normal weight range (*p* = 0.0001) (Figure 2).

**Figure 3:**
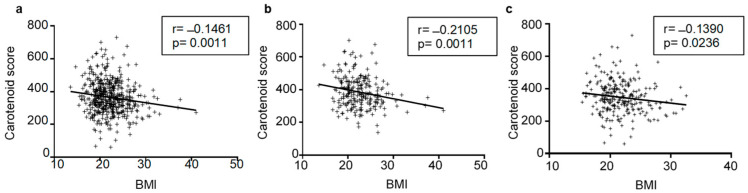
Correlations between carotenoid score and BMI in the total population (**a**), males (**b**), and females (**c**). The association between the carotenoid content and BMI was analyzed using Pearson’s correlation test. For each linear regression graph, the correlation coefficient—r and the statistical significance—*p* are reported.

In addition, we found an inverse correlation between BMI and the carotenoid score in the total population (a) (r = −0.1461, *p* = 0.0011), males (b) (r = −0.2106, *p* = 0.0011), and females (c) (r = −0.1390, *p* = 0.0236) (Figure 3).

Using a multiple regression adjusted model, the association among the carotenoid score and BMI, age, and sex was evaluated in the total population. We confirmed that BMI and sex had a significant association with the carotenoid score (*p* < 0.0001 and *p* < 0.0001, respectively), while age had no impact on this association (Table 2).

### 3.3. Adherence to the Mediterranean Diet Based on Food Questionnaires

**Figure 4:**
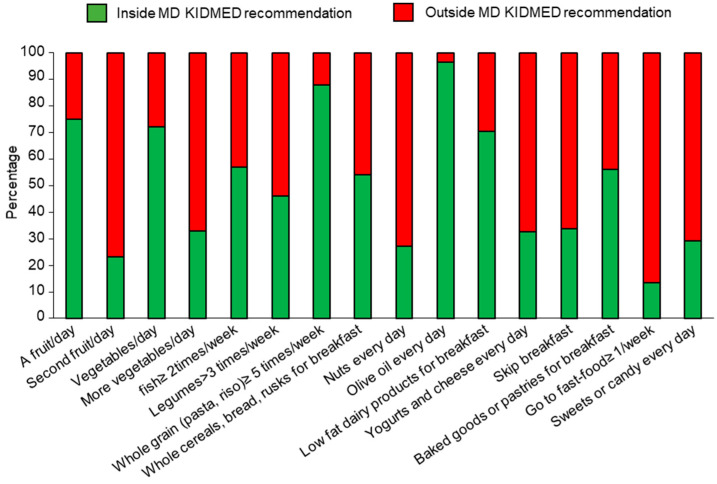
Compliance with items from the KIDMED test in the total sample. Frequency (%) of population distribution with respect to the cut-off points within or outside recommendations according to the KIDMED score.

In order to evaluate the adherence to the MD in our sample population, we administered the KIDMED and the MD Pyramid tests. The compliance rates for each MD recommendation using the KIDMED questionnaire in the total population were calculated and shown in Figure 4. Analyzing the population stratified by sex, no significant differences in the food rates for most of the dietary recommendations between males and females were found (Supplementary Table S1).

Using the KIDMED score, an average degree of adherence to the MD (5.43 ± 2.57) was found in the total population, with males showing significantly higher values compared to females (5.72 ± 2.46 vs. 5.17 ± 2.63, *p* = 0.0168). In addition, a significantly increased percentage of females compared to males was observed within the low adherence group (28% vs. 17%, *p* = 0.0029) (Table 3).

**Figure 5:**
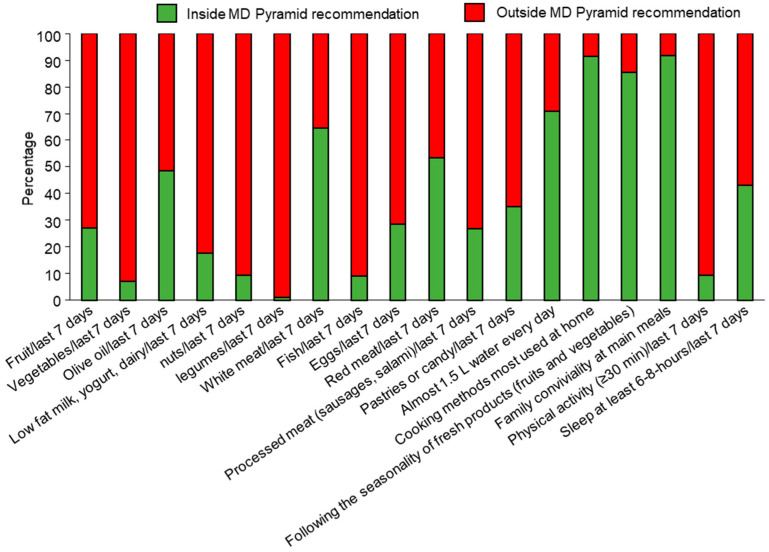
Graphical representation of the MD Pyramid questionnaire in the total population with respect to the cut-off points inside or outside recommendations.

Similar results were obtained using the MD Pyramid test. The frequency of each MD recommendation obtained via the administration of the MD Pyramid questionnaire in the total population is represented in Figure 5. Stratifying the total population according to sex, a significantly higher consumption of olive oil consumption in the last 7 days (56.23% vs. 39.91%, *p* = 0.023) and white meat intake in the last 7 days (73.58% vs. 54.08%, *p* = 0.003) were found in females than in males. In contrast, daily water consumption (84.98% vs. 58.49%, *p* < 0.0001) and practice physical activity for at least 30 min in the last 7 days (13.73% vs. 5.28%, *p* = 0.03) were greater in males than in females (Supplementary Table S2).

Similar to the results obtained using the KIDMED test, the mean adherence of the total population evaluated with the MD Pyramid test fell in the medium range (7.20 ± 1.93). No statistically significant differences were observed when dividing adolescents by gender (Table 4).

### 3.4. Correlation between the Mediterranean Diet Scores and Skin Carotenoid Content

**Figure 6:**
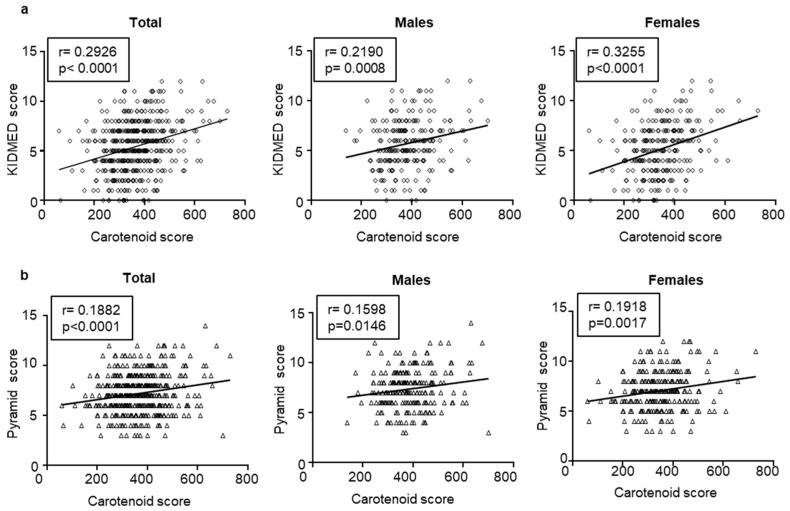
Correlations between carotenoid content and KIDMED (**a**) and MD Pyramid score (**b**) in the total population or categorized by sex. The association between carotenoid content and MD adherence was calculated using Pearson’s correlation test. For each linear regression graph, the correlation coefficient—r and the statistical significance—*p* are reported.

Skin carotenoid content represents an important biomarker for FV intake. As expected, we observed that the skin carotenoid content is positively associated with the frequency of FV intake evaluated by KIDMED and MD Pyramid questionnaires in our sample population (Supplementary Tables S3–S6). Interestingly, we found a positive correlation between the KIDMED score and the carotenoid content (a) in the total population (r = −0.2926, *p* < 0.0001), males (r = −0.2190, *p* = 0.0008), and females (r = −0.3255, *p* < 0.0001) (Figure 6a). The same direct correlation between the MD adherence and the carotenoid content was observed using the MD Pyramid score (b) in the total population (r = −0.1882, *p* < 0.0001), males (r = −0.1598, *p* = 0.0146), and females (r = −0.1918, *p* = 0.0017) (Figure 6b).

In addition, classifying the population according to their sex, we found significantly higher carotenoid content via KIDMED in males than in females of the assessed medium (380.70 ± 87.54 vs. 340.45 ± 92.87, *p* = 0.0007) adherence group. Similarly, we showed that the carotenoid score was higher in males than in females via the MD Pyramid test in the low (379.72 ± 97.33 vs. 330.49 ± 103.10, *p* = 0.003) and medium (385.47 ± 90.81 vs. 358.99 ± 94.18, *p* = 0.04) adherence groups (Table 5).

## 4. Discussion

In this study, we used the Veggie Meter^®^ as a non-invasive tool for the detection of the skin carotenoid content in a population of healthy Calabrian adolescents, and we demonstrated for the first time, to our knowledge, that the carotenoid score was positively associated with the adherence to the MD pattern. The MD represents one of the healthiest eating and lifestyle patterns worldwide based on the high consumption of local and seasonal products of plant origin, including FV. It has been widely demonstrated that good adherence to the MD decreases the development and progression of several metabolic and chronic-degenerative diseases, including type 2 diabetes, obesity, cardiovascular and neurodegenerative diseases, metabolic syndrome, and different types of cancer [10], and improves the quality of life [42,46]. To date, adherence to the MD is assessed via dietary scores based on food consumption questionnaires.

Here, using the KIDMED and the MD Pyramid tests as two different MD pattern-consistent and pattern-inconsistent food consumption questionnaires, we found a medium MD adherence in our sample population. These findings are in agreement with the results from our and other studies in which adolescents living in the Mediterranean regions of Southern Italy showed average adherence to the MD [42,43]. In our work, we observed a higher adherence to the MD in the male than in the female population. In contrast, Morelli et al. have reported that the medium MD adherence evaluated using the KIDMED test for the total adolescent sample population of Southern Italy was independent of sex [42]. In the same geographical area, no gender-related differences were found in MD adherence in an adult population of Southern Italy [48]. Similarly, Raparelli et al. have demonstrated that sex did not impact MD adherence in an adult population with ischemic heart disease [49]. It has been demonstrated that optimal adherence to the MD has a positive effect on health, preventing the development of non-communicable diseases [8,46,50,51]. Augimeri et al. have found that adolescents with optimal adherence to the MD had higher estimated consumption of polyphenols calculated by FV intake and displayed a better serum lipid profile with respect to adolescents consuming low polyphenol amounts [8]. Interestingly, the serum from subjects who optimally adhered to MD recommendations was able to prevent lipid accumulation in hepatic cells and to reduce the production of proinflammatory cytokines in inflammatory macrophages in vitro with respect to lower MD adherers, supporting the importance of enhancing the adherence to the MD pattern toward an optimal compliance [8,51]. Thus, campaigns to promote MD adherence are needed, especially in the young population, in order to prevent the development of chronic degenerative diseases in adulthood.

Although it has been widely demonstrated that dietary scores are validated tools that establish adherence to the MD, the heterogeneity of the MD adherence scores highlights the importance of developing innovative methods for a more accurate interpretation of MD adherence. Based on the MD recommendations, daily FV consumption is suggested to provide an adequate intake of micronutrients. To date, the intake of FV is evaluated by self-reported FV intake or by measuring the blood carotenoid levels via an invasive and expensive liquid chromatography/mass spectrometry-based analytic method [23,52]. The use of the Veggie Meter^®^ has been already described as a validated and non-invasive method for skin carotenoid detection, which accurately reflects the FV intake [25,33,34,53]. Therefore, it represents an important tool for an objective measurement of FV intake to overcome bias associated with self-reported questionnaires, allowing us to easily analyze the FV intake in community-based settings. According to the data reported in the literature, we found that the content of the skin carotenoids was positively associated with the FV intake evaluated using both the KIDMED and the Pyramid tests in our adolescents.

Interestingly, we found a mean carotenoid score of 364.75 in our sample population, which is within the normal range and directly related to MD adherence. However, the carotenoid score in our adolescents was higher compared to that observed in the American adolescent population, potentially due to an increased FV consumption [54]. Furthermore, we highlighted significantly higher values of skin carotenoids in the male compared to the female population. Conflicting data are present in the literature regarding the correlation between carotenoid levels and gender. In fact, some authors did not detect a gender difference in the carotenoid score [34,55], whereas Obana et al. reported higher carotenoid levels in females than in males [56]. Further studies are needed in order to understand whether gender plays a significant role in the accumulation of and variation in the skin carotenoid content and to establish a clearer relationship between gender and carotenoid status. Although the body’s carotenoid content depends on their dietary intake, some anthropometric parameters and lifestyle habits can influence their accumulation in the tissues. In agreement with data present in the literature [55], in our study, we identified a negative correlation between the carotenoid score and BMI, which was also confirmed in a multiple regression model with age and sex. Collectively, we demonstrated, for the first time, that the skin carotenoid content measured with the Veggie Meter^®^ is positively associated with adherence to the MD evaluated via two MD food questionnaires, suggesting that it represents a promising tool to assess MD adherence.

This study has several limitations. All adolescents were volunteers and enrolled without exclusion criteria. We also did not formally ask about health problems, drug use, and any type of restrictive diet or digestive problems, which could impair the absorption of carotenoids because this was not part of the ethical approval. Finally, some FV do not contain carotenoids, and thus, the Veggie Meter^®^ failed in their detection.

## 5. Conclusions

The use of the Veggie Meter^®^ is a new non-invasive method to measure the skin carotenoid content directly related to the adherence to MD. This portable instrument, which allows for a fast and reliable analysis of the FV intake, can be used to perform screening and promotion campaigns for healthy lifestyles in order to spread the principles of the Mediterranean model, particularly in the young population, and prevent the onset of metabolic and chronic degenerative diseases in adults.

## Figures and Tables

**Table 1:** General characteristics and carotenoid content by Veggie Meter^®^ measurement in the total population, males, and females.

Characteristics	Total	Males	Females	*p*-Value
Subject number	498	233	265	
Age (years)	16.66 ± 1.53	16.54 ± 1.57	16.77 ± 1.48	0.25
Weight (Kg)	62.87 ± 12.51	68.91 ± 13.05	57.57 ± 9.21	**<0.0001**
Height (cm)	168.32 ± 8.78	174.58 ± 6.70	162.81 ± 6.36	**<0.0001**
BMI (Kg/m^2^)	22.10 ± 3.44	22.55 ± 3.67	21.71 ± 3.18	**0.0001**
Z-score	0.39 ± 4.50	0.31 ± 0.99	0.10 ± 0.90	**0.03**
Carotenoid Score	364.75 ± 98.29	384.52 ± 94.17	347.37 ± 98.72	**<0.0001**

BMI: body mass index.

**Table 2:** Multiple regression analysis models with carotenoid score and BMI, age, and sex.

	β	se	CI (95%)	*p*-Value
BMI	−5.176	1.261	−7.654, −2.697	**<0.0001**
Age	4.451	2.831	−1.112, 10.014	0.117
Sex = M	−42.535	8.644	−59.519, −25.552	**<0.0001**
Adjusted R^2^		0.0637		

β: regression coefficient, se: standard error, CI: confidence interval, M: males.

**Table 3:** Stratification of the total sample divided by sex with respect to the Mediterranean diet adherence evaluated via KIDMED test.

MD	Total	Males	Females	*p*-Value
Low (<4)	n = 113 (23%)	n = 39 (17%)	n = 74 (28%)	**0.0029**
Medium (4–7)	n = 283 (57%)	n = 143 (61%)	n = 140 (53%)	0.0548
High (>7)	n = 102 (20%)	n = 51 (22%)	n = 51 (19%)	0.4658
KIDMED score	5.43 ± 2.57	5.72 ± 2.46	5.17 ± 2.63	**0.0168**

n: number of subjects.

**Table 4:** Adherence to the Mediterranean diet based on MD Pyramid test.

MD	Total	Males	Females	*p*-Value
Low (<7)	n = 189 (38%)	n = 81 (35%)	n = 108 (41%)	0.1692
Medium (7–12)	n = 308 (62%)	n = 151 (65%)	n = 157 (59%)	0.2024
High (>12)	n = 1 (0.2%)	n = 1 (0.4%)	n = 0 (0%)	0.2857
Pyramid score	7.20 ± 1.93	7.36 ± 1.95	7.05 ± 1.91	0.0741

**Table 5:** Adherence to the Mediterranean diet based on KIDMED or MD Pyramid test.

		Carotenoid Content			
	Score	Total Sample	Males	Females	*p*-Value
KIDMED	Low (<4)	332.12 ± 83.91	356.56 ± 83.45	319.24 ± 81.79	0.07
	Medium (4–7)	360.79 ±92.29	380.70 ± 87.54	340.45 ± 92.87	**0.0007**
	High (>7)	411.91 ± 111.68	416.63 ± 111.32	407.20 ± 112.95	1
Pyramid	Low (<7)	351.59 ± 103.33	379.72 ± 97.33	330.49 ± 103.10	**0.003**
	Medium (7–12)	371.97 ± 93.33	385.47 ± 90.81	358.99 ± 94.18	**0.04**
	High (>12)	631	631	-	

## Data Availability

The data presented in this study are available in this article and the Supplementary Material.

## Supplementary Materials

The following supporting information can be downloaded at: https://www.mdpi.com/article/10.3390/nu15234920/s1, Table S1: Frequency (%) of each recommendation in the population divided by sex based on the KIDMED test; Table S2: Frequency (%) of each recommendation in the population divided by sex based on the Pyramid test; Table S3: The carotenoid content in the total population or categorized by gender with respect to the fruit consumption based on KIDMED test; Table S4: The carotenoid content in the total population or categorized by gender with respect to the vegetable consumption based on KIDMED test; Table S5: The carotenoid content in the total population or categorized by gender with respect to the fruit consumption/7 days based on Pyramid test; Table S6: The carotenoid content in the total population or categorized by gender with respect to the vegetable consumption/7 days based on Pyramid test.

## Appendix A

**Table A1:** Mediterranean Diet Pyramid questionnaire.

Questions	Criteria for 1 point
In the last 7 days, how many times have you eaten fruits?	1–2 times/day
In the last 7 days, how many times did you eat vegetables (cooked or raw as salads)?	≥2 times/day
In the last 7 days, how many times have you used extra virgin olive oil to season your dishes?	Every day
In the last 7 days, how often did you drink milk or eat yogurt and dairy products?	2 times/day
In the last 7 days, how many times have you eaten dried fruit?	1–2 times/day
In the last 7 days, how many times did you eat legumes?	Every day
In the last 7 days, how many times have you eaten white meat?	1–2 times in the last 7 days
In the last 7 days, how many times have you eaten fish?	≥2 times in the last 7 days
In the last 7 days, how many times have you eaten eggs?	2–4 times in the last 7 days
In the last 7 days, how many times have you eaten red meat?	<2 times in the last 7 days
In the last 7 days, how many times have you eaten processed meat (cured meats and sausages)?	≤1 times in the last 7 days
In the last 7 days, how many times have you eaten sweets (snacks, biscuits, croissants, etc.)?	≤2 times in the last 7 days
Do you usually consume at least 1.5 L of water as your main drink every day?	Yes
What are the most used cooking methods at home for preparing food?	Steaming, boiling, oven
Do you usually consume foods according to the seasonaliy of fresh products (fruit and vegetable) or do you consume foods without worrying about their seasonality?	Yes
During main meals (lunch and dinner) does the family gather or is it not a frequent habit?	Yes
In the last 7 days, how many times did you practice physical activity for 30 min or more?	Every day
In the last 7 days, how many times did you sleep for at least 6–8 h?	Every day
