# Supplementary material for: Association between Skin Carotenoid Score Measured with Veggie Meter® and Adherence to the Mediterranean Diet among Adolescents from Southern Italy
Source: Nutrients. 2023 Nov 25;15(23):4920. doi: 10.3390/nu15234920 (PMC10707776; doi:10.3390/nu15234920)
Supplement: Supplementary file 1 [file nutrients-15-04920-s001.zip › nutrients-2709452-supplementary.pdf]

Supplementary Table S1. Frequency (%) of each recommendation in the population divided by sex based on the KIDMED test.

KIDMED items	Males	Females	*p*-value
a fruit/day	75.11	74.72	1
second fruit/day	26.18	20.38	0.31
vegetables/day	69.10	74.72	0.34
more vegetables/day	27.04	38.11	0.10
fish ≥ 2 times/week	62.23	52.08	0.15
legumes> 3 times/week	49.36	43.40	0.39
whole grain (pasta, riso)≥ 5 times/week	91.42	84.91	0.13
whole cereals, bread, rusks for breakfast	58.80	49.81	0.20
nuts every day	28.76	25.66	0.63
olive oil every day	95.71	96.98	0.70
low fat dairy products for breakfast	74.68	66.42	0.16
yogurt and cheese every day	34.76	30.57	0.55
skip breakfast	76.82	56.98	**0.003**
baked goods or pastries for breakfast	40.77	46.42	0.47
go to fast-food≥1/week	87.55	85.66	0.67
sweets or candy every day	74.25	67.92	0.34

Supplementary Table S2. Frequency (%) of each recommendation in the population divided by sex based on the Pyramid test.

Pyramid items	Males	Females	*p*-value
fruits/last 7 days	32.19	22.64	0.154
vegetables/last 7 days	6.01	7.92	0.579
olive oil/last 7 days	39.91	56.23	**0.023**
low fat milk, yogurt, dairy/last 7 days	21.46	14.34	0.193
nuts/last 7 days	11.16	7.92	0.469
legumes/last 7 days	1.72	0.38	0.561
white meat/last 7 days	54.08	73.58	**0.003**
fish/last 7 days	11.16	7.17	0.323
eggs/last 7 days	35.19	22.64	0.061
red meat/last 7 days	50.21	56.23	0.395
processed meat (sausages, salami)/last 7 days	23.61	29.43	0.423
pastries or candy/last 7 days	33.91	36.23	0.767
almost 1,5 L water every day?	84.98	58.49	**<0.0001**
cooking methods most used at home	92.27	90.57	0.800
following the seasonality of fresh products (fruit and	84.98	85.66	0.841
family conviviality at main meals	92.27	91.32	0.800
physical activity (≥30 min)/last 7 days	13.73	5.28	**0.03**
sleep at least 6-8 hours/last 7 days	47.21	39.25	0.073

Supplementary Table S3. The carotenoid content in the total population or categorized by gender with respect to the fruit consumption based on KIDMED test.

KIDMED test			
**Fruit consumption**	**Total**	**Males**	**Females**
1 fruit/day	377.15±99.17	391.20±97.57 (175)	364.46±99.14 (198)
2 fruits/ day	399.23±105.13	414.18±104.58	381.75±104.035
***p*-Value**	**0.0401**	0.114	0.266

Supplementary Table S4. The carotenoid content in the total population or categorized by gender with respect to the vegetable consumption based on KIDMED test.

**KIDMED test**	**Carotenoid score**		
**Vegetable consumption**	**Total**	**Males**	**Females**
1 vegetable/day	380,25±99,56	401,64±96,29	362,47±98,98
2 vegetables/day	401,63±101,10	410,95±102,34	395,67±100,37
***p*-Value**	**0.024**	0.524	**0.0070**

Supplementary Table S5. The carotenoid content in the total population or categorized by gender with respect to the fruit consumption/7 days based on Pyramid test.

**Pyramid test**	**Carotenoid score**		
**Fruit consumption/ 7 days**	**Total**	**Males**	**Females**
no consumption	310.31±83.77	350.45±50.01	289.29±90.97
1-2/week	336.27±94.12	370.31±90.08	310.26±89.14
3-6/week	373.16±89.86	379.93±91.64	367.8±88.58
1-2/day	391±95.54	398.79±96.72	381.27±93.95
3+/day	466.23±137.35	459.25±131.39	477.4±161.65
***p*-Value**	§**0.98**, ***0.0001**, ¥ **<0.002**, ~ **0.0001**	§ ns, * ns, ¥ ns, ~ **ns**	§ ns, ***0.0005**, ¥ **0.002**, ~ **0.0001**

§no consumption *vs* 1-2 week; *no consumption *vs* 1-2 day; ¥ no consumption *vs* 3-6 week; ~ no consumption *vs* 3+ day

Supplementary Table S6. The carotenoid content in the total population or categorized by gender with respect to the vegetable consumption/7 days based on Pyramid test.

	**Pyramid test**		
**Vegetable consumption/ 7 days**	**Total**	**Males**	**Females**
no consumption	295.87±57.72	304.5±64.01	283.92±47.52
1-2/week	341.55±94.18	376.66±89.46	311.33±87.91
3-6/week	365.65±95.96	390.01±85.5	344.6±99.91
1-2/day	388.78±91.37	402.51±97.26	376.55±84.67
3+/day	436±112.47	422.57±128.05	444.95±103.13
***p*-Value**	§0.05, ***0.0006**, ¥ **0.0001**,~ **0.0001**	§ **0.01**,* **0.0004** ¥ **0.002**, ~ **0.001**	§ ns, * 0.004 ¥ 0.09,~ **0.0001**

§no consumption *vs* 1-2 week; *no consumption *vs* 1-2 day; ¥ no consumption *vs* 3-6 week; ~ no consumption *vs* 3+ day
